# Prevalence of *Escherichia coli* and *Salmonella* spp. in Colombian Pig Production Settings: A One Health Perspective Study

**DOI:** 10.3390/vetsci13020189

**Published:** 2026-02-14

**Authors:** Adriana Pulido-Villamarín, Fidson-Juarismy Vesga, Camilo Venegas, Deyci Rodríguez-Cordero, Adriana Matiz-Villamil, Irina Barrientos, Iliana C. Chamorro-Tobar, Juan Pablo Caicedo, Beatriz Ariza, Seyli Gomez, Loti Sarai Bermudez, Ana Karina Carrascal-Camacho, Moises Aranda-Silva, David Olaya E

**Affiliations:** 1 Agricultural Research Unit-UNIDIA, Department of Microbiology, Faculty of Sciences, Pontificia Universidad Javeriana, Bogotá 110231, Colombia; seyli.gomez@javeriana.edu.co; 2 Center for Research and Technology Transfer of the Pig Sector—Ceniporcino, Asociación Porkcolombia-Fondo Nacional de la Porcicultura, Bogotá 110231, Colombia; ibarrientos@porkcolombia.co (I.B.); ichamorro@porkcolombia.co (I.C.C.-T.); 3 Faculty of Veterinary Sciences, Universidad Nacional del Centro de la Provincia de Buenos Aires (UNICEN), Tandil 7000, Argentina; 4 Microbiological Quality of Water and Sludge Laboratory, Environmental and Industrial Biotechnology Research Group-GBAI, Department of Microbiology, Faculty of Sciences, Pontificia Universidad Javeriana, Bogotá 110231, Colombia; vesga.f@javeriana.edu.co (F.-J.V.); c.venegas@javeriana.edu.co (C.V.); caicedotjuanp@javeriana.edu.co (J.P.C.); bermudez.loti@javeriana.edu.co (L.S.B.); 5 Food Microbiology, Environmental and Industrial Biotechnology Research Group-GBAI, Department of Microbiology, Faculty of Sciences, Pontificia Universidad Javeriana, Bogotá 110231, Colombia; deyci.rodriguez@javeriana.edu.co (D.R.-C.); acarrasc@javeriana.edu.co (A.K.C.-C.); 6 Applied Biotechnology Laboratory, Environmental and Industrial Biotechnology Research Group-GBAI, Department of Microbiology, Faculty of Sciences, Pontificia Universidad Javeriana, Bogotá 110231, Colombia; amatiz@javeriana.edu.co; 7 Clinical Laboratory Sciences Group, Hospital Universitario San Ignacio, Bogotá 110231, Colombia; deariza@husi.org.co; 8 Department of Mathematics, Faculty of Sciences, Pontificia Universidad Javeriana, Bogotá 110231, Colombia; maranda@javeriana.edu.co; 9 Institute of Public Health, Pontificia Universidad Javeriana, Bogotá 110231, Colombia; d.olaya@javeriana.edu.co

**Keywords:** Colombia, *Escherichia coli*, One Health, *Salmonella* spp., swine production

## Abstract

Zoonotic pathogens in swine production can negatively impact both human and animal health, with the environment serving as a potential transmission vehicle. Therefore, this study aimed to determine the prevalence of *Escherichia coli* and *Salmonella* spp. in the Colombian swine production chain using the One Health approach. Samples were collected from nine farms and two slaughterhouses in the departments of Antioquia, Cundinamarca, Valle del Cauca, and Meta. The analyzed samples included water, feed, pig and worker feces, organic material in treatment (manure treated and compost), and pig carcasses. These samples were analyzed using standard microbiological methods and the Molecular Detection System (MDS). The results showed *Salmonella* spp. prevalence rates of 15.47% in pigs, 9.4% in feed, 8.47% in water, and 2.56% in organic material. For *E. coli* O157, prevalence rates were 25.71% in pigs, 10% in feed, 22.22% in water, and 33.33% in organic material. The high prevalence and bacterial loads in water suggest it is a critical reservoir and a potential primary source of contamination in the production chain. Although these pathogens were not detected in workers, the zoonotic risk remains. Additionally, the prevalence of haemolytic enterotoxigenic *E. coli* (ETEC), a major swine pathogen, was 40.1%. This study emphasizes the need to improve biosecurity and farm management practices to reduce the risk of environmental transmission, thereby minimizing public, occupational, and animal health risks. Implementing water treatment protocols and improving organic waste management are recommended to limit the spread of bacterial contamination. These actions are based on the ‘One Health’ approach, recognizing that animal health and ecosystem integrity are indivisible pillars of human health.

## Simple Summary

There are bacteria, known as zoonotic bacteria, that affect the health of animals and humans, and the environment can be a potential transmission vehicle. These bacteria can be present on pig farms and in slaughterhouses. We determined the presence of *Escherichia coli* and *Salmonella* spp. in nine Colombian farms and two slaughterhouses. Using laboratory techniques, these bacteria were detected in samples of water, feed, feces from pigs and workers, treated manure/compost, and pig carcasses. The results showed the presence of *Salmonella* spp. in between 2.56% and 15.47% of the samples, while *E. coli* O157 was present in between 10% and 33.33%. The high presence of these bacteria in the water suggests that it may be the source of contamination throughout the production chain. Although these pathogenic bacteria were not detected in workers, the zoonotic risk exists. This study highlights the need to improve biosecurity practices and farm management to reduce the risk of environmental transmission, thereby lowering risks to public, occupational, and animal health. Water treatment protocols and improved organic waste management are recommended to reduce bacterial contamination. These actions are based on the One Health approach, recognizing that animal health and environmental stability are essential for good human health.

## 1. Introduction

Pork is one of the animal-derived foods that significantly enhances the human diet, especially in developing countries [1]. The Organization for Economic Co-operation and Development (OECD) and the Food and Agriculture Organization (FAO) anticipate an 11% increase in global pork consumption by 2032 [2]. Specifically, Colombia’s per capita consumption is projected to reach 15 kg/year by 2024, with an annual growth rate of 6.2% [3]. Antioquia, Cundinamarca, Valle del Cauca, and Meta are the leading pork-producing regions, where a pig population of nearly 6,000,000 head of livestock is concentrated and where the farms are type-technified [4].

Gastrointestinal infections caused by pathogens such as *Salmonella* spp. and *Escherichia coli* can negatively impact swine production and public health. These infections result in economic losses in production [5] and represent a zoonotic risk to farm workers’ health and consumers’ safety [5,6], and additionally, they are carriers of antibiotic-resistant genes [7,8].

The genus *Salmonella* includes two species, *S. bongori* and *S. enterica*. The latter comprises six subspecies and approximately 2600 serovars and is generally pathogenic to warm-blooded animals [9]. Similarly, *E. coli* is characterized by over 200 serovars and several pathotypes, each associated with specific virulence factors. Enterotoxigenic *E. coli* (ETEC) and Shiga toxin-producing *E. coli* (STEC) are of particular importance. ETEC is a major concern for swine health, whereas STEC, including *E. coli* O:157, is a critical public health concern [10,11].

Zoonotic pathogens can be found in the pig production system throughout the entire production chain, from primary production through the pre-slaughter phase in slaughterhouses. These pathogens are major contributors to foodborne diseases (FBD) and waterborne diseases (WBD), which have a significant impact on global health within the framework of the “One Health” approach. This integrative approach underscores the interconnectedness of human, animal, and environmental health and leverages these relationships to develop innovative disease surveillance and control strategies [12].

Given that comprehensive multi-matrix studies in Colombia that simultaneously evaluate the production chain, the environmental context, and the human component under the ‘One Health’ approach are still limited, this study aims to close critical information gaps to mitigate health risks in the country. In this context, the aim of this study was to determine the prevalence of *E. coli* and *Salmonella* spp. in swine populations (primary production and slaughterhouses), farm workers, water, animal feed, and manure/compost across nine swine farms and two slaughterhouses in Colombia’s four central pork-producing regions.

## 2. Materials and Methods

### 2.1. Ethical Approvals

The procedures for collecting animal-derived samples were approved by the Animal Welfare Committee of the Faculty of Veterinary Sciences, Universidad Nacional del Centro de la Provincia de Buenos Aires (Animal Welfare Act ResCA 087/02). Human-related procedures and research protocols were authorized by the Research and Ethics Committee of the Faculty of Sciences at Pontificia Universidad Javeriana (Act No. 5/23).

Participation by adult pig farm workers was voluntary, with confidentiality guaranteed and informed consent obtained in accordance with ethical guidelines. Workers who had received antibiotic treatment within 15 days before recruitment or were undergoing treatment during the study period were excluded.

### 2.2. Study Design

A one-year, exploratory cross-sectional study using simple random sampling was conducted at a single time point.

### 2.3. Study Population

#### 2.3.1. Farms

Nine technified commercial farms, defined as having ≥100 sows and ≥600 fattening pigs, voluntarily participated in the study. This classification follows guidelines from the Colombian Agricultural Institute (ICA) [13]. The farms were located in Antioquia (n = 4), Cundinamarca (n = 3), Valle del Cauca (n = 1), and Meta (n = 1). Samples collected from the farms included swine and human feces, swine drinking water, swine feed, and in-process organic material (manure/compost) (OMUP). Sampling focused on specific production cycle phases, including replacement sows, pregnant sows, lactating sows, nursery or sucking piglets, and growing and finishing pigs.

The number of samples from pigs was calculated at a 95% confidence level, a 5% margin of error, and an expected prevalence of 30%. The size calculated was 320 samples, distributed according to the total population of each farm.

For environmental samples from each farm, these were selected randomly in five for each type by age stadium, plus a water entrance, and three for OMUP.

#### 2.3.2. Slaughterhouse

Two slaughterhouses in Antioquia and Cundinamarca voluntarily participated in the study. Samples collected included caecal contents and carcass swabs.

The samples were selected randomly, 20 per sample type in each slaughterhouse.

### 2.4. Sample Collection

Before sample collection, participating farms held sensitization workshops, and all necessary materials, including personal protective equipment (PPE), were provided.

The names of the farms and human participants were anonymized in accordance with data protection law. All samples were labeled with a consecutive laboratory number.

#### 2.4.1. Pig Farm Workers’ Feces

After receiving technical instructions, the voluntary participants collected their fecal samples by natural defecation. The samples were placed in airtight, wide-mouth containers.

#### 2.4.2. Pig Feces

Fresh pooled fecal samples were collected from the pen floor. The central portion of at least five fresh droppings was obtained, avoiding the surface layer, which may be contaminated by flies, and the lower layer in direct contact with the ground. The pooled sample was placed in a hermetically sealed bag for transport and analysis.

#### 2.4.3. Water Samples

Samples were collected from the primary water source supplying the farm and from nipple drinkers in the pens housing pigs of various age groups. The samples were collected in sterile 1 L wide-mouth containers, filled to about 1% air space.

#### 2.4.4. Feed Samples

A total of 500 g of feed was collected from feeders or troughs in the pens, with samples differentiated by age group. Samples were placed in sterile, hermetically sealed bags. If multiple feeders were present in the pen, 250 g were collected from each and pooled.

#### 2.4.5. OMUP (Organic Material Under Treatment)

Samples were collected from liquid manure tanks and/or several compost bins. Pooled final compost samples (500 g) were stored in hermetically sealed bags.

#### 2.4.6. Processing Slaughterhouse Samples

Carcass samples were collected using a non-destructive swabbing method. A sterile sponge moistened with 15 mL of buffered peptone water (ISO; Neogen) was used to swab defined 100 cm^2^ areas of the cranial region (mandibular area), ventral abdominal region (belly), dorsal lumbar region (loin), and pelvic limb region (leg) [14,15]. Caecal content was collected by using a sterile scalpel to cut the cecum and transferring approximately 50 mL of its contents into a sterile bag [16].

All samples were transported in hermetically sealed, refrigerated containers. The transport time ranged from 2 to 8 h, depending on the location of the farm or slaughterhouse.

### 2.5. Sample Processing

#### 2.5.1. Pig Farm Worker’s Feces

Fecal samples were processed according to protocols adapted from the Laboratory Surveillance Guide for Acute Diarrheal Diseases [17] and the Foodborne Disease Outbreak Surveillance Protocol [18], with modifications developed by the Hospital Universitario San Ignacio (HUSI) in Bogotá, Colombia. Briefly, stool cultures were performed on selective and differential media for *E. coli* O157 (MacConkey agar and MacConkey sorbitol agar) (Scharlau; Scharlab S.L., Sentmenat, Spain), and selective enrichment for *Salmonella* spp. was carried out in Rappaport and selenite broths, followed by isolation on Hektoen agar (Scharlau; Scharlab S.L., Sentmenat, Spain) and CHROMagar™ Salmonella (CHROMagar Microbiology, Paris, France). Plates were incubated at 35 °C for 24 to 48 h. Genus and species were identified by mass spectrometry (Vitek®MSTM, bioMérieux, Montreal, QC, Canada).

#### 2.5.2. Non-Human Matrices (MDS Method)

Pig feces, water, feed, OMUP, carcass swabs, and caecal contents samples were processed using the presence/absence method with the Molecular Detection System (MDS) and AOAC^®^ methods 2017.01 and 2016.01 (OMA), based on loop-mediated isothermal amplification (LAMP) technology [19]. Detection kits were used to detect *E. coli* O157 and *Salmonella* spp. (100% Inclusivity, 100% Exclusivity).

A total of 100 mL of each water sample was filtered through 0.45 µm nitrocellulose membrane filters (Millipore®, Darmstadt, Germany) [20]. The membranes were then transferred to 90 mL of ISO-buffered peptone water. Additionally, 25 g of porcine fecal matter, feed, OMUP, and caecal contents were added to 225 mL of ISO-buffered peptone water. The sponge used to collect carcass samples was returned to the original sampling bag. Then, 35 mL of ISO-buffered peptone water was added to reach a final volume of 50 mL.

All samples suspended in ISO-buffered peptone water were incubated at 42 °C for 24 h. Then, the protocol provided by the respective test kit manufacturer was followed.

#### 2.5.3. Cultural Confirmation and Additional Analysis

Following detection with the 3M^®^ Molecular Detection System(Neogen® Lansing, MI, USA), samples testing positive for *E. coli* O157 were further analyzed by culturing on MacConkey agar and MacConkey sorbitol agar (Scharlau; Scharlab S.L., Sentmenat, Spain). Colonies exhibiting characteristic phenotypes, such as lactose-positive and sorbitol-negative (colorless), were cryopreserved for further analysis. Additionally, porcine fecal samples were cultured on MacConkey agar and blood agar (Scharlau; Scharlab S.L., Sentmenat, Spain)with 5% sheep blood to evaluate haemolytic activity typical of *E. coli* ~ ETEC.

For samples that tested positive for *Salmonella* spp., 100 µL of ISO-buffered peptone water was inoculated into 10 mL of Muller-Kauffmann Tetrathionate broth and Rappaport-Vassiliadis broth (Scharlau; Scharlab S.L., Sentmenat, Spain). These enrichment broths were incubated at 42 °C for 24 h. Subsequently, isolation was carried out on Hektoen agar (Scharlau; Scharlab S.L., Sentmenat, Spain) and CHROMagar™ Salmonella Plus agar (CHROMagar Microbiology, Paris, France). Characteristic colonies—H_2_S-positive with a transparent halo on Hektoen agar and mauve-pink on CHROMagar™ agar—were cryopreserved for further analysis.

#### 2.5.4. Fecal Contamination Indicators (FCI) in Water Samples

Quantification of total coliforms (TC) and *E. coli* was performed using the ISO 9308-1 [21] membrane filtration method and reported as CFU/100 mL. Filters were plated on Chromocult agar (Merck, Darmstadt, Germany)and incubated at 35 (±2) °C. Colonies exhibiting dark blue/purple staining were identified as *E. coli*, and the total number of red colonies and *E. coli* was recorded as TC. Positive controls included *E. coli* ATCC^®^25992 and *Klebsiella pneumoniae* ATCC^®^700603; *Salmonella enterica* ATCC^®^13076 was used as a negative control.

### 2.6. Data Analysis

Data were processed in Excel to determine means, maximum and minimum values, and frequency indices, which were expressed as percentages. Visualizations were created using Flourish|Data Visualization and Storytelling. The data were also analyzed using the chi-square tests (with Monte Carlo simulations) and proportion tests.

## 3. Results

### 3.1. Overall Prevalence

A total of 896 samples were collected, distributed in 367 fecal samples from pigs of various age groups (lactating sows, piglets, nursery pigs, and fattening pigs), 189 water samples (from natural sources and nipple drinkers in pens housing pigs of different age groups), 181 feed samples supplied to the studied age groups, 39 samples of organic matter in process (manure treated or compost) (OMUP), 24 samples from farm workers, and 92 samples collected in slaughterhouses (46 carcass swabs and 46 samples of cecal contents).

Nationwide, the prevalence of *Salmonella* spp. was 15.5% in pigs (primary production and slaughterhouses), 9.4% in feed, 8.5% in water, and 2.6% in OMUP. For *E. coli* O:157, the prevalence was 25.7% in pigs, 10% in feed, 22.2% in water, and 33.3% in OMUP. None of the pathogens were detected in samples from farm workers.

### 3.2. Departmental Variation

**Figure 1:**
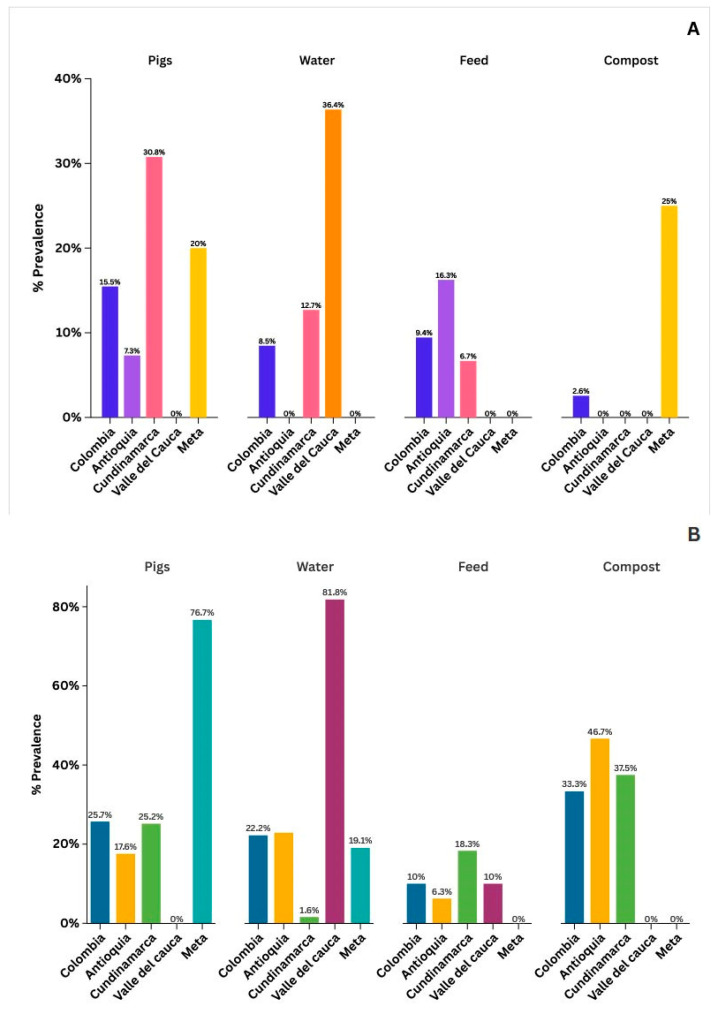
Prevalence of *Salmonella* spp. (**A**) and *E. coli* O157 (**B**) on farms across Colombian departments.

The department with the highest *Salmonella* spp. positivity in pigs was Cundinamarca (30.8%), while the highest prevalence in water was observed in Valle del Cauca (36.4%), and in feed in Antioquia (16.3%). A statistically significant association was found between bacterial presence and department (*p* < 0.001). Similarly, for *E. coli* O:157, a significant association with the department was observed (*p* < 0.001). The highest prevalence in pigs was found in Meta (76.7%), in water in Valle del Cauca (81.8%), and in feed and OMUP in Cundinamarca (18.3% and 40%, respectively). Detailed data by department are presented in Figure 1.

### 3.3. Variation in the Stages of the Production Cycle

**Figure 2:**
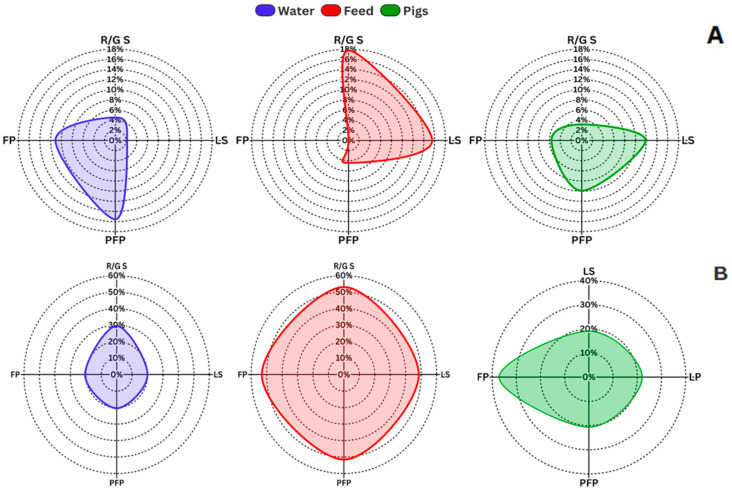
Prevalence of *Salmonella* spp. (**A**) and *E. coli* O157 (**B**) across the different age groups analyzed. R/G S: Replacement/Gestation Sows, LS: Lactating Sows, PFP: Pre-Fattening Piglets, and FP: Fattening pigs.

By contrast, *Salmonella* spp. was present in 7.6% (28/367) of pigs on farms (primary production) and in 50% (46/92) of carcasses at slaughterhouses. Similarly, *E. coli* O157 was detected in 26.2% (96/367) of pigs from farms and 26.1% (24/92) of carcasses at the slaughterhouse. Data by age group are shown in Figure 2.

Due to its clinical implications, *E. coli* (Haemolytic~ETEC) was prevalent in 40.1% of the swine population across the farms. Its characterization was previously published [22].

Furthermore, *E. coli* biotype 1 was found in 77.7% of the water samples, 66.7% of the OMUP samples, and 75% of the human population samples.

### 3.4. Fecal Indicators in Water/Feed

**Figure 3:**
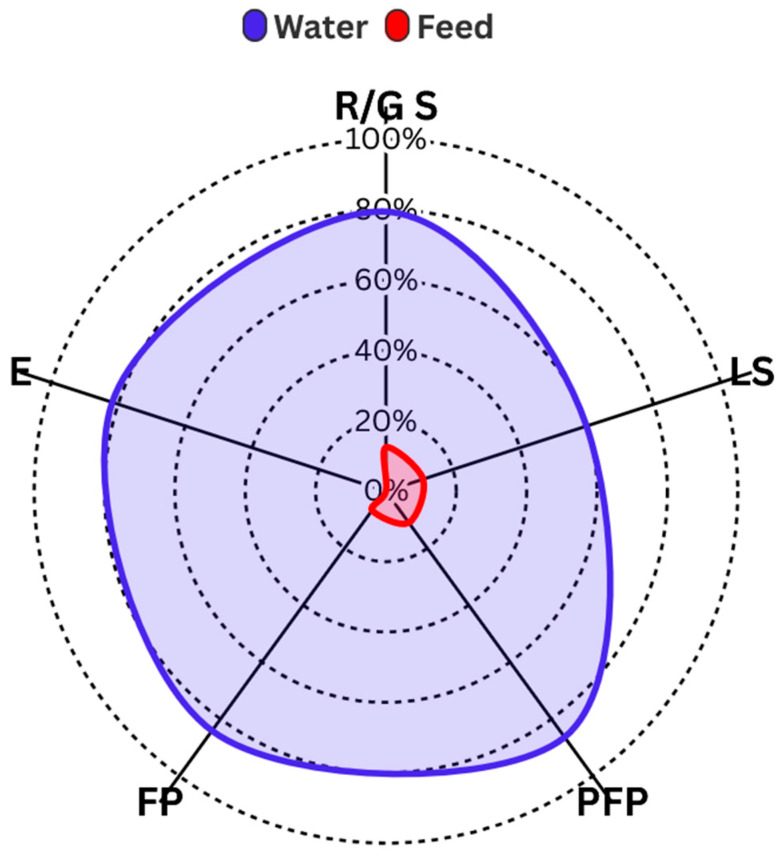
Prevalence of *E. coli* in the different age groups analyzed in water and feed matrices. R/G S: Replacement/Gestation Sows; LS: Lactating Sows; PFP: Pre-Fattening Piglets; FP: Fattening pigs; E: environment.

Indicators of fecal contamination in water showed a high prevalence of *E. coli* across all assessed groups: 79.5% in Replacement/Gestation Sows (R/G S), 60% in Lactating Sows (LS), 86.4% in Pre-Fattening Piglets (PFP), 84.1% in Fattening Pigs (FP), and 81.8% in the Environment (E) (Figure 3). A comparison of the two proportions shows that the prevalence of *E. coli* in feed was considerably lower: with 12.5% in R/G S feed (*p* < 0.00, CI: 0.621-100%), 11.1% in LS (*p* < 0.00, CI: 0.324-100%) and PFP feed (*p* < 0.00, CI: 0.615-100%), and 6.7% in FP feed (*p* < 0.00, CI: 0.642-100%) (Figure 3).

Because each analyzed department is geographically distant and affected by different environmental factors related to its water sources, the water quality parameters are presented separately.

**Figure 4:**
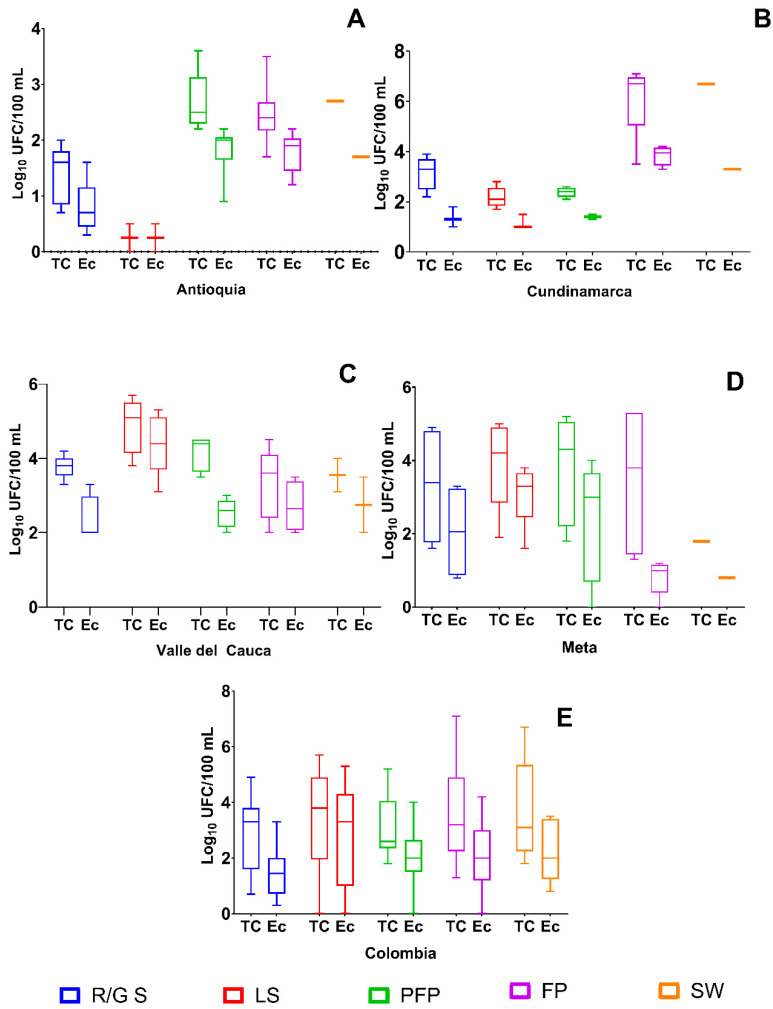
Concentrations of Total Coliforms (TC) and *E. coli* (Ec) in Log_10_ CFU/100 mL across departments and matrices for various age groups. R/G S: Replacement/Gestation Sows; LS: Lactating Sows; PFP: Pre-Fattening Piglets; FP: Fattening Pigs; SW: Source Water. (**A**) In Antioquia; (**B**) In Cundinamarca; (**C**) Valle del Cauca; (**D**) Meta; (**E**) Colombia Total.

In Antioquia, the highest average concentrations of total coliforms (CT) were recorded at the Pre-Fattening Piglets (PFP) site (mean: 2.7; range: 2.2–3.6 Log_10_ CFU/100 mL), followed by the Fattening Pigs (FP) stage (2.5; 1.7–3.5). The Source Water (SW) site showed a stable and slightly lower value (2.7), while Lactating Sows (LS) had a mean CT of 0.2 (0.0–0.5), and the lowest values were found in Replacement/Gestation Sows (R/G S), with an average of 1.4. For *E. coli*, FP and PFP had the highest means (1.8), while LS and R/G S had notably lower levels (0.2 and 0.8, respectively), suggesting reduced fecal contamination in early reproductive stages (Figure 4A).

In Cundinamarca, both SW and FP sites had the highest CT concentrations, with means of 6.7 and 6.1, respectively, and relatively narrow ranges. These elevated and consistent values indicate persistent microbial contamination. R/G S and LS showed moderate values (3.1 and 2.2), while PFP registered the lowest (2.4; 2.1–2.6). For *E. coli*, the highest mean was detected in FP (3.9), with lower values across the other stages, particularly in R/G S (1.4), supporting a contamination gradient by production stage (Figure 4B).

In Valle del Cauca, the highest CT concentrations were recorded in the LS (4.9; 3.8–5.7) and PFP (4.2; 3.5–4.5) stages. SW and FP sites also showed considerable contamination, with mean CT values of 3.6 and 3.3, respectively. R/G S had slightly higher levels (3.8), indicating consistent microbial presence across stages. *E. coli* concentrations peaked in LS (4.4), while the lowest mean was observed in SW (2.7), suggesting substantial fecal contamination in piglets and reproductive stages (Figure 4C).

In Meta, TC concentrations were highest in R/G S and PFP (3.3 and 3.8, respectively), with broad ranges indicating sample variability. LS also showed elevated values (3.9; 1.9–5.0), while the lowest TC was found in SW (1.8), with no variation. The *E. coli* profile followed a similar trend, with the highest levels observed in LS (3.1) and R/G S (2.1), and the lowest again in SW (0.8), highlighting localized sources of fecal pollution (Figure 4D).

At the national level (Colombia), SW sites had the highest mean TC concentration (3.7), followed by FP (3.6), LS (3.3), and R/G S (2.9). The lowest mean was observed in PFP (3.1), though values varied by region. For *E. coli*, the highest mean was reported in LS (2.8), while the lowest was in SW (2.3). These data highlight the roles of both the production stage and site in determining microbial quality, with implications for targeted water management strategies and pig health (Figure 4E).

## 4. Discussion

Pathogens such as *Salmonella* spp. and *E. coli* O157, which are highly relevant to the One Health approach, are commonly detected in swine production systems worldwide. In Colombia, previous studies have reported the presence of *Salmonella* spp. at various stages and matrices in the swine production chain. For instance, average prevalence rates of 8.2% in pigs at the primary production stage (based on fecal and rectal swabs) and 14.3% in water from nipple drinkers in pens have been documented [23,24,25], which are consistent with the results of this study. However, the 0.3% prevalence reported in animal feed by Barrientos et al. (2018) [26] contrasts with the higher prevalence observed in this study. Additionally, prevalence in pork carcasses shows significant variability, with a combined prevalence of 9.7% reported in slaughterhouses [27], which is substantially lower than the 43.5% (20/46) detected in this study.

Clinically, *Salmonella* can occur at any stage. However, its presence in lactating and weaned piglets can worsen post-weaning diarrhea. In Canada, prevalence rates of 43%, 29%, and 28% have been reported in lactating sows, nursery pigs, and growing-finishing pigs, respectively [28]. These rates are notably higher than those in this study and align with other national findings [23]. These findings may reflect the implementation of biosecurity measures on Colombian farms. However, even low prevalence may be due to contamination from water and feed, which are considered significant risk factors for *Salmonella* on farms [23,25,28]. Additionally, asymptomatic *Salmonella* carriers in lactating sows can transmit the pathogen to piglets during this stage [29].

Although *E. coli* O157 does not pose a clinical risk to swine health, pigs can serve as carriers, potentially contaminating the environment, especially when organic waste management on farms is inadequate. Such contamination can impact water quality and contribute to air pollution [30]. This is Colombia’s first report of its detection in the swine production chain.

In contrast, the presence of *E. coli* ETEC has significant clinical implications for swine, as it is the primary pathogen responsible for neonatal and post-weaning diarrhea, resulting in high morbidity and mortality rates [7,31]. In Mexico, molecular analysis detected a prevalence of 9.6% [32]. These figures are considerably lower than the 40% prevalence reported in this study, which was determined based on phenotypic characteristics, including β-hemolysis, and confirmed through Api20e^®^ galleries (Biomérieux™, Canada).

Reports on the prevalence of *E. coli* O157 in pork carcasses are limited. However, Nastasijevic et al. (2020) in the U.S. and Essendoubi et al. (2000) in Canada found a 5.4% and 1.4% of *E. coli* STEC, respectively, during the chilling phase in pork slaughterhouses [33,34]. These findings emphasize the role of scalding and chilling processes in reducing pathogen presence and, consequently, the risk of transmission to humans through pork-based products [35,36].

Results for total coliforms (TC) and *E. coli* concentrations showed notable variation across production stages and regions. The highest concentrations were observed in pre-fattening piglets (PFP) and in the Meta and Valle del Cauca regions. In contrast, the lowest *E. coli* levels were recorded in Cundinamarca (Figure 3 and Figure 4). This geographic variability in water microbiological quality suggests that local management practices and environmental conditions are influential factors [37].

The high prevalence of *E. coli* in water used during production contrasts with the significantly lower prevalence in feed. These findings highlight water as a possible primary vehicle for contamination, necessitating stricter control measures, including water treatment schemes to remove turbidity and suspended solids. Similar studies in Serbia by Milanov et al. (2020) reported TC concentrations of up to 2.7 × 10^3^ CFU/100 mL and *E. coli* at 1.2 × 10^1^ CFU/100 mL in well water samples [38].

The higher prevalence of *E. coli* in water underscores the need to enhance biosecurity measures, especially in phases with high-density rearing and intensive handling, where stress increases the risk of pathogen transmission. Lekagul et al. [39] reported that careful management prevents piglet diseases. In addition, Münster and Kemper (2024) [40] evaluated drinking water for animals in Germany and found that 47.4% of the samples exceeded the microbiological parameters, indicating a potential risk to pig health and highlighting the need to adopt effective hygiene measures to prevent the transmission of microorganisms through water and to maintain animal health and the safety of food products.

The prevalence of *E. coli* (10%) and *Salmonella* spp. (9.4%) in pig feed across the studied Colombian farms was higher than the 6.1% and 4.3% previously reported by ICA between 2018 and 2021 [41]. Although feed shows lower prevalence than water, it remains a critical vehicle for pathogens and resistance genes, posing a risk of gastrointestinal infections throughout the swine production chain. Contamination likely occurs through cross-contamination in feeding systems and surfaces during rearing, or via environmental exposure during storage to reservoirs such as wild birds and domestic animals—known sources of *E. coli* O157. These pathways facilitate pathogen movement between animal and human reservoirs [42,43], highlighting the need for targeted control strategies at specific contamination points to mitigate zoonotic transmission.

From an environmental perspective, the results of this study present an intriguing finding, compared to Matiz et al. [44], who reported no detection of *Salmonella* spp. or *E. coli* O157. This discrepancy may indicate the possible presence of antibiotics that are frequently administered at subtherapeutic doses through feed or drinking water, resulting in animal waste laden with high levels of antimicrobials. Applying these materials to agricultural soil, even after composting or converting them into organic fertilizers, introduces substantial quantities of these substances into the environment. Specifically, antimicrobials may leach from the soil into the underlying surface and groundwater, leading to environmental contamination and associated risks to human and animal health [45]. This implies that the manure deposition from swine production systems is recognized as a potential source of AMR spread [46].

This study did not detect *Salmonella* spp. *or E. coli* O157 in fecal samples from farm workers. This contrasts with reports from Uganda, where non-typhoidal *Salmonella* was found in 6.6% (8/122) of workers, and serotyping revealed a variety of serovars. Moreover, 50% of the serovars detected in slaughtered pigs were also identified in slaughterhouse workers [47], underscoring the significant role of pigs as reservoirs of zoonotic pathogens. Although *Salmonella* was not detected in humans in the present study, the risk of zoonotic contamination remains relevant. Supporting this, a recent study in Palestine found genetically identical strains of *S. enterica* in chicken manure, chicken meat, and asymptomatic *poultry workers*, highlighting the potential for silent transmission *throughout* the food chain [48]. Hence, regardless of the livestock production system, contact with farm animals can be recognized as a risk factor for pathogen transmission [49,50]. Studies show that educational interventions for slaughterhouse workers significantly improve knowledge, hygiene practices, and proper meat handling, resulting in safer products and reduced occupational risks. This highlights the need for specialized training programs focused on hygiene and *behavior* among farm and slaughterhouse workers [51,52].

This study provides crucial baseline data on the prevalence of zoonotic pathogens across the Colombian swine production chain, highlighting critical control points such as water management. Future studies incorporating molecular typing would be valuable to elucidate direct transmission routes. Additionally, these pathogens pose a significant concern due to their antimicrobial resistance, as noted by the WHO [52,53]. Future research should also aim to identify their AMR genes, especially since tetracycline (TC), amoxicillin (AMOX), norfloxacin (NOR), ampicillin (AMP), and imipenem (IMP) are recognized as emerging environmental contaminants related to AMR, particularly in water [53].

## 5. Conclusions

*Salmonella* spp. was detected in 15.5% of pigs (primary production and slaughterhouses), 9.4% of feed samples, 8.5% of water samples, and 2.6% of organic material. *E. coli* O157 showed a prevalence of 25.7% in pigs, 10% in feed, 22.2% in water, and 33.3% in organic material. The presence of Enterotoxigenic *E. coli* (Haemolytic ~ ETEC), important in swine health, was detected in 40.1%. In swine workers, *Salmonella* spp. and *E. coli* O:157 were not detected; only *E. coli* was found in 75%.

The high prevalence of *E. coli* O157 and *Salmonella* spp. in water, particularly during the gestation and grow-finish phases in Valle del Cauca, underscores the role of water as a possible source of fecal contamination. To mitigate this risk, it is imperative to prioritize strategic hygiene and water treatment interventions at all production stages to mitigate the transmission of these bacteria and ensure food safety in the sector.

The presence of zoonotic pathogens underscores the need to improve biosecurity and management practices on farms to reduce the potential for environmental transmission, thereby minimizing risks to public health, occupational health, and animal health.

## Data Availability

The original contributions presented in this study are included in the article. Further inquiries can be directed to the corresponding author.
